# Integrated genomic analysis identifies clinically relevant subtypes of renal clear cell carcinoma

**DOI:** 10.1186/s12885-018-4176-1

**Published:** 2018-03-13

**Authors:** Peng Wu, Jia-Li Liu, Shi-Mei Pei, Chang-Peng Wu, Kai Yang, Shu-Peng Wang, Song Wu

**Affiliations:** 1 The Affiliated Luohu Hospital of Shenzhen University, Department of Urological Surgery, Shenzhen University, Shenzhen, 518000 China; 2 Shenzhen Following Precision Medical Institute, Shenzhen Luohu Hospital Group, Shenzhen, 518000 China; 3 Shenzhen Second People’Hospital, 1st affiliated hospital of ShenZhen University, Shenzhen, 518037 China; 4 College of Basic Medical Sciences, Dalian Medical University, Dalian, 116044 China

**Keywords:** ccRCC, Gene expression, Molecular classification, Pathway

## Abstract

**Background:**

Renal cell carcinoma (RCC) account for over 80% of renal malignancies. The most common type of RCC can be classified into three subtypes including clear cell, papillary and chromophobe. ccRCC (the Clear Cell Renal Cell Carcinoma) is the most frequent form and shows variations in genetics and behavior. To improve accuracy and personalized care and increase the cure rate of cancer, molecular typing for individuals is necessary.

**Methods:**

We adopted the genome, transcriptome and methylation HMK450 data of ccRCC in The Cancer Genome Atlas Network in this research. Consensus Clustering algorithm was used to cluster the expression data and three subtypes were found. To further validate our results, we analyzed an independent data set and arrived at a consistent conclusion. Next, we characterized the subtype by unifying genomic and clinical dimensions of ccRCC molecular stratification. We also implemented GSEA between the malignant subtype and the other subtypes to explore latent pathway varieties and WGCNA to discover intratumoral gene interaction network. Moreover, the epigenetic state changes between subgroups on methylation data are discovered and Kaplan-Meier survival analysis was performed to delve the relation between specific genes and prognosis.

**Results:**

We found a subtype of poor prognosis in clear cell renal cell carcinoma, which is abnormally upregulated in focal adhesions and cytoskeleton related pathways, and the expression of core genes in the pathways are negatively correlated with patient outcomes.

**Conclusions:**

Our work of classification schema could provide an applicable framework of molecular typing to ccRCC patients which has implications to influence treatment decisions, judge biological mechanisms involved in ccRCC tumor progression, and potential future drug discovery.

**Electronic supplementary material:**

The online version of this article (10.1186/s12885-018-4176-1) contains supplementary material, which is available to authorized users.

## Background

ccRCC, the most common type of kidney cancer, representing approximately 92% of such cases. Most people with kidney cancer are usually over 55 years of ages and this cancer is more common in men [1].The global pattern of genetic changes underlying ccRCC includes alterations in genes controlling cellular oxygen sensing and the maintenance of chromatin states [2]. Early mutations and inactivation of VHL is commonly seen in ccRCC [3]. Other recurrently mutated genes include PBRM1, BAP1 and SETD2, located in chromosome 3p, whose loss is the most frequent arm-level events inccRCC (91% of samples) [4]. Losses on chromosome 14q and gains of 5q were also frequent observed, specially, the former is associated with more aggressive phenotype [5]. These genetic aberrations are critical for clinical diagnosis and personal therapy. We collected gene expression data of ccRCC from TCGA and using Consensus Clustering [6] algorithm cluster all samples to detect potential subtypes. We discovered three subtypes and survival analysis showed one subclass has far poorer prognosis than the other three. Thus, we compared the poor subclass with the other and find some pathways changes and genes that may cause adverse outcomes.

## Methods

### Consensus clustering identified three subtypes of ccRCC

Features of Consensus Clustering algorithm are the 2D feature and item subsampling and it provides a method to represent the consensus across multiple runs of a clustering algorithm, to determine the number of clusters in the data, and to assess the stability of the discovered clusters [6]. The method can also be used to represent the consensus over multiple runs of a clustering algorithm with random restart (such as K-means, model-based Bayesian clustering, SOM, etc.), so as to account for its sensitivity to the initial conditions [7].This method has gained popularity in cancer genomics, where new molecular subclasses of disease have been discovered [8–11].

**Fig. 1:**
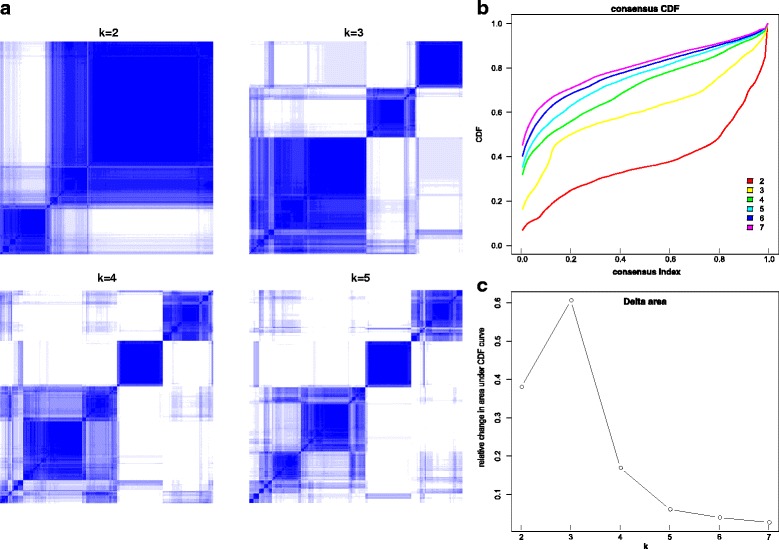
**a** Consensus matrices. Both rows and columns represent samples and consensus values range from 0(never clustered together) to 1 (always clustered together) marked by white to dark blue. The cluster memberships are marked by colored rectangles. **b** Consensus Cumulative Distribution Function (CDF) Plot. CDF plot shows the cumulative distribution functions of the consensus matrix for each k (indicated by colors) **c** Delta Area Plot. This graphic shows the relative change in area under the CDF curve. In k = 3, the shape of the curve approaches the ideal step function, and shape hardly changes as we increase K past 3

**Fig. 2:**
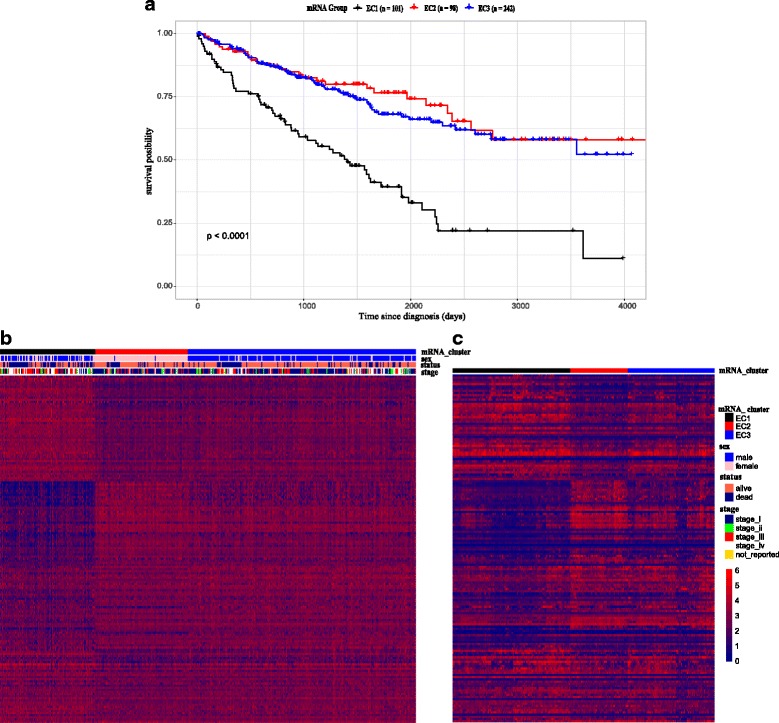
**a** Kaplan-Meier Overall Survival Curves. survival plot by Kaplan-Meier method, EC1 has worse prognosis compared with the other. **b** The heatmap of ccRCC expression data. Using consensus clustering algorithm, samples are classified into three types. The heatmap shows that EC1 subtype has higher mortality and more patients in stage III, IV than the other groups

We select the samples of ccRCC in expression data which contain molecular subtypes in TCGA and filter out samples without molecular information and genes with low signal across samples to get more precise classification results. We classify samples into three robust expression clusters (EC) utilizing Consensus Clustering together with hierarchical clustering. The clustering stability increases from k = 2 to k = 3, but not for k > 3 (Fig. 1a and b) and delta area under the curve in k = 3 also has appreciable increase (Fig. 1c). Combined with the clinical data, we performed Kaplan meier analysis and the survival curve shows the EC1 subtype are obviously more malignant than the other (Fig. 2a).

### Validation of subtypes in an independent data set

To validate our results, an independent data set including 265 ccRCC patients from GEO was used to assess the subtype reproducibility [12]. We visualize the expression data by a 164 classifying marker gene list and hierarchical clustering. The marker genes were identified in EC1–3 subtypes by combining Wilcoxon signed-rank test and permutation (see methods). Unsurprisingly, the validation data set almost coincided with the data set of TCGA, comprising of three subtypes (Additional file 1: Figure S1) and representing similar expression profile (Fig. 2b and Fig. 2c). Considering differences in sample size and different sequencing techniques, obvious concordance was seen between our classification and the results from the earlier study, which further proves the reliability of our analysis and the authenticity of the three subtypes.

### Genetic aberrations and Clinicopathological parameters of subtypes

We classified the three types into malignant and relative unmalignant types because of survival analysis results which shows EC2–3 has approximate prognosis. We summarized the copy number mutations and single nucleotide variation of EC1 and top five recurrent mutation genes are VHL, PBRM1, MUC4, BAP1 and SETD2 (42.57%, 25.74%, 20.79%, 19.80%, 13.86%). Frequency of frequently mutated genes except BAP1 were similar between these two types (Table 1), but the high-level somatic copy number variation (SCNV) regions between two groups were quite different (Table 2). BAP1, a nuclear deubiquitinase, is inactivated in 15% of ccRCCs. A significant increase in BAP1 mutation frequency was observed in EC1compared with the remainder of the samples, which is consistent with BAP1 is a potential tumor suppressor and relevant to bad outcome in ccRCC [13, 14].

**Table 1:** Mutation frequency of genes with single nucleotide variations in two groups

Genes	EC1	EC2–3	*P* values	All
VHL	42.57%	54.41%	0.0480	51.70%
PBRM1	25.74%	35.35%	0.0948	33.10%
MUC4	20.79%	18.25%	0.3941	18.82%
BAP1	19.80%	5.89%	4.508e-05	9.07%
SETD2	13.86%	12.93%	0.9421	13.15%

**Table 2:** Known cancer genes in the high-level copy number variation regions

	EC1	Known cancer related genes in Region	EC2–3	Known cancer related genes in Region
High-level amplified events				
Cytoband	5q35	FGFR4/DOCK2 (9.09%)	5q35	FGFR4/DOCK2 (19.94%)
	5q32	CD74/CSF1R (9.09%)	5q31	CTNNA1/NR3C1 (17.86%)
	5q33	PDGFRB/ZNF300 (9.09%)	5q33	PDGFRB/ZNF300 (17.86%)
High-level deletion events				
Cytoband	9p21	CDKN2A/CDKN2B (11.11%)	3p25	PPARG/RAF1/VHL (15.18%)
	9p23	PTPRD (7.07%)	3p21	PBRM1/SETD2/BAP1 (15.18%)
	3p25	PPARG/RAF1/VHL (6.06%)	3p22	TGFBR2/MYD88 (14.58%)

Gain of 5q paired with loss of 3p was observed frequent in both EC1 and EC2–3 when it is a highly frequent event in ccRCC. However, loss of 9q21 presents a higher frequency in EC1 and three common tumor suppressor genes (TSG) are deleted in this area including CDKN2A, CDKN2B and MTAP. CDKN2A and CDKN2B act as tumor suppressors by regulating the cell cycle which block traversal from G1 to S-phase or inhibits cell cycle G1 progression. The deletion, mutation or promoter methylation of the two genes are common in various cancers, which help to the unlimited growth of cancer cells and CDKN2A is associated with metastatic cancer [15–18]. MTAP is key enzyme in the methionine salvage pathway and frequently deleted in human cancers because of its chromosomal proximity to CDKN2A [19]. This SCNA pattern may conduce to increase the potential of proliferation for EC1 subtype.

The clinical and pathological features are largely distinct from each other (Table 3). We compare EC1 with EC2–3 in the two datasets from four perspectives: age, gender, grade and stage and estimate the significance by chi-squared test of 2*2 table. Besides gender, EC1 is highly interrelated with older age, advanced grade and stage in TCGA data, which partly explains the result that this subtype has a poor prognosis. In GEO data, the results are similar although the Pvalues are not statistically significant enough, probably due to the unavailable patient information. We will discuss these results detailly in the following analysis.

**Table 3:** Clinical characteristics of subtypes

		TCGA data			GEO data		
		EC1	EC2–3	*P* values	EC1	EC2–3	*P* values
Age (mean ± SD)		63.5 ± 10.9	60.2 ± 12.5	0.01168	NA	NA	NA
Gender	male	66	224	1	81	79	0.04636
	female	35	116		38	64 (3NA)	
Pathological grade	Grade 1 + 2	20	174	4.67e-08	43	69	0.05186
	Grade 3 + 4	81	166		74(2NA)	70 (7NA)	
Stage	Stage I–II	35	220	1.478e-07	22	31	0.09323
	Stage III-IV	66	120		42 (55NA)	30 (85NA)	

### Enrichment analysis reveals high potential of EC1 in proliferation and metastasis

**Fig. 3:**
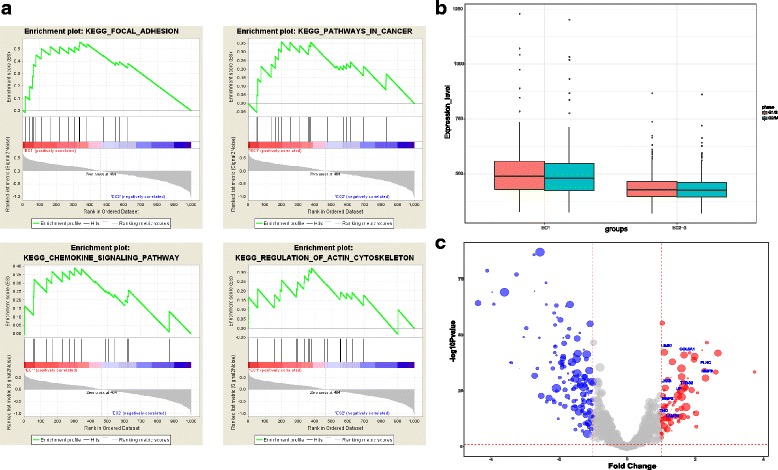
**a** Enrichment plot of upregulation pathways in EC1**.** GSEA of expression data from EC1 441 with worse prognosis, as compared to EC2–3. X-axis is the enrichment score of each gene. Y-axis represents the order of the gene in dataset. **b** Volcano plot of differential genes. Red color: up-regulated in EC1. blue color: down-regulated in EC1. Grey: not differential genes. Size of the bubble: mean expression of each gene C box plot of mean expression level on G1/S and G2/M gene set. EC1 is higher than EC2–3

In order to reveal the statistically significant, concordant differences between EC1 and other subtypes, gene sets enrichment analysis (GSEA) algorithm (see methods) is used and we chose KEGG gene sets as predefined gene sets [20]. Consequently, 7 gene sets are upregulated in EC1 and 5 gene sets are upregulated in EC2–3. The 7 gene sets of EC1 mainly focus on cell proliferation and mobility containing Focal Adhesion, Regulation of Actin Cytoskeleton and Chemokine (Fig. 3a). These pathways implicate epithelial-to-mesenchyme transition (EMT), cell proliferation and migration, closely related to tumor progression and metastasis. By contrast, up-regulated pathways in EC2–3 are mainly involved in metabolism including PPAR signaling pathway and Cytochrome P450. Next, our analysis concentrates on the core enrichment genes, which contributes to the leading-edge subset within the predefined gene set and have high expression level, in up-regulated pathways of EC1 for its poor prognosis (see methods).

Actin Cytoskeleton pathway is significantly enriched and there are 12 genes among core enrichment (Additional file 2: Table S1). MAPK Signal pathway is enriched with 15 genes, but only FLNC is core enrichment gene (Additional file 3: Table S2). It is noteworthy that FLNC is also core enrichment gene in Focal Adhesion pathway (Additional file 4: Table S3) and it has been reported that FLNC can be a potential progression marker for the development of hepatocellular carcinoma [21]. LAMB3 is core enrichment gene in Focal Adhesion and Pathways in Cancer (Additional file 5: Table S4) and research shows that repressing LAMB3 inhibit mutant KRAS-Driven tumor growth [22]. LAMB3 is also associated with EMT, a crucial change that happens to cancer cells before metastasis, and several researches conclude that high expression of LAMB3 is correlated with tumor metastasis including oral squamous cell carcinoma, bladder cancer and breast cancer [23–25]. Other core enrichment genes in Pathways in Cancer including MMP9 and MMP2, together with LAMB3, which are components of the extracellular matrix, may be considered as a molecular biomarker for ccRCC progress and metastasis. Chemokine Signaling Pathway containing CCL5, CXCL9 and CCR5 are also upregulated in EC1 (Additional file 6: Table S5). Chemokines play an important role in tumor growth and angiogenesis, on the one hand, providing cytokines to promote tumor growth, on the other hand, improving matrix metalloproteinase activity, promoting tumor cell through the cell membrane so as to increase the probability of tumor metastasis. These features reveal that EC1 subtype are more invasive and has a higher likelihood of migration, indicating that patients with EC1 expression pattern are prone to distant metastasis, resulting in poor prognosis. To evaluate the proliferation ability of subtypes, we next scored each sample for the expression signatures for the G1/S and G2/M phases. EC1 has higher expression level than EC2–3 (C1/S: Ttest Pvalue = 9.486514e-07, G2/M: Ttest Pvalue = 1.371606e-06), which reflects, to some extent, that the subtype has a stronger proliferative capacity (Fig. 3b). Moreover, we perform differential genes analysis between EC1 and EC2–3 to evaluate the significance of expression difference and find 11 genes that upregulated in 4 pathways of EC1 are differential genes (Padj< 0.01 & |log2FoldChange| > 1). These genes are all high expressed in EC1 subtype (Fig. 3c).

### Intratumoral gene interaction network in EC1 relates to cell adhesion and motion

After obtaining the relative enrichment pathways in EC1, we aim to investigate the intratumoral gene interaction network. Weighted correlation network analysis (WGCNA) algorithm (see methods) was employed to detect gene interaction modules and intramodular hub genes in EC1 subtype [26]. Seven major modules are detected and the gene co-expression pattern within these modules was very high (Additional file 7: Figure S2, Additional file 8: Figure S3). After modules were detected, we performed enrich analysis on the Seven modules we identified and blue colored module is markedly enriched with genes in pathways implicating Cell adhesion molecules, Cytokine-cytokine receptor interaction, Regulation of Actin Cytoskeleton and Chemokine Signaling Pathway (Table 4). This result, consistent with previous analysis, demonstrate that these pathways are interrelated with each other and may lead to a poor outcome for patients, which can be used as a monitoring indicator of cancer progression.

**Table 4:** Enrich pathways in blue module

Blue Module					
Description	Genes in Gene Set (K)	Genes in Overlap (k)	k/K ratio	*p*-value	FDR q-value
Cell adhesion molecules(CAMs)	134	9	0.0672	1.06E-08	1.97E-06
Regulation of actin cytoskeleton	216	10	0.0463	5.56E-08	5.17E-06
Chemokine signaling pathway	190	9	0.0474	2.14E-07	1.33E-05
Cytokine-cytokine receptor interaction	267	10	0.0375	3.97E-07	1.82E-05
Primary immunodeficiency	35	5	0.1429	4.90E-07	1.82E-05
Leukocyte transendothelial migration	118	7	0.0593	1.10E-06	3.41E-05
Natural killer cell mediated cytotoxicity	137	7	0.0511	2.99E-06	7.94E-05
Complement and coagulation cascades	69	5	0.0725	1.50E-05	3.49E-04
Hematopoietic cell lineage	88	5	0.0568	4.88E-05	1.01E-03
Jak-STAT signaling pathway	155	6	0.0387	7.36E-05	1.37E-03
Toll-like receptor signaling pathway	102	5	0.049	9.86E-05	1.67E-03

Hub genes are defined as genes that interacted with other genes most. Correlation between modules and clinical data reveals the blue module, comprised of 200 genes, demonstrating delicacy correlation with tumor stage (Additional file 9: Figure S4), may play a fatal part in the growth and metastasis of ccRCC. For the blue module, JAK3 is the hub gene filled with red color, accompanying by LIMK1 and DENND2D (Additional file 10: Figure S5), which are also hub genes but with less connectivity. JAK3 is a well-known cancer gene, playing an important role in tumorigenesis and progression of hematological malignancy [27] especially in leukemia [28, 29] and JAK3 inhibitor has been applied into treating for autoimmune and blood cancer in clinic. LIMK1, a critical regulator of actin dynamics, functioning as a regulatory role in tumor cell invasion and proliferation [30], has been reported in gastric and lung cancer [31–33]. Further studied are needed to investigate the role of JAK3 and LIMK1 in the development of ccRCC.

### Discrepancies in methylation levels of genes contribute to different phenotypes

**Fig. 4:**
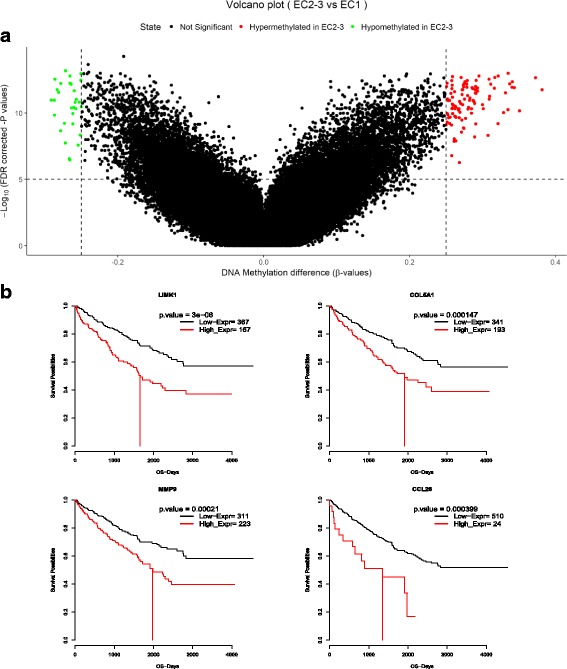
**a** Volcano plot of differential methylation sites. Data are obtained from HM450K methylation data. β-values represent mean methylation level of CpG sites. **b** Kaplan meier survival plot of four genes. Red line indicates the median survival time

To gain insights into the methylation states of pathways and genes, we explored the methylation levels for EC1 and EC2–3 group. HM450K data of ccRCC was used and unexpectedly, higher overall methylation levels are observed in EC1 (Pvalue = 3.7e-06) (Additional file 11: Figure S6). We inferred that the distinctions of methylation in specific genomic region result in the changes in expression pattern. Thus, we calculate the difference for each probe between the mean DNA methylation of each group and test for differential expression using Wilcoxon test adjusting by the Benjamini-Hochberg method to search for differentially methylated CpG sites. 93 significant hypomethylation CpG sites were found in EC1 subgroup (absolute beta-values difference 0.2 & Padj < 0.01) and 60 are located in the protein coding region (Fig. 4a). 6 genes are related to Focal Adhesion and cell adhesion molecular in EC1 including DOCK1, LAMC1 and TLN1. We also observed epigenetic silencing of TOLLIP in EC1 and TOLLIP deficiency is associated with decreased T-cell responses [34], which may reflect the immune suppression phenomenon in EC1.

To analyze the connection between the genes that we identify involving Focal adhesion and cell adhesion molecular and prognosis of patients, we perform survival analysis and divide patients into two parts (high expression group: Zscore > 1.96 and low expression group: Zscore < 1.96, confidence interval = 95%) according to the expression level of genes. We find that most of the genes that enrichment or hypomethylation in EC1 are negatively related to prognosis. Four genes involving in Focal Adhesion, Pathway in cancer, Chemokine and cytoskeleton are relevant to survival evidently including LIMK1, COL5A1, MMP9 and CCL26 (Fig. 4b).

## Results

We use the consensus clustering method and discover a new subpopulation of ccRCC, with a poor prognosis, higher degree of malignancy, pathological grade and clinical stage. The features of the subgroup in gene mutation, expression interation network and methylation manifest stronger potential of proliferation and metastasis, coinciding with the clinical performance which furtherly validate our findings.

## Discussion

Here, we apply unsupervised Consensus Clustering algorithm and identify three distinct subtypes based on hierarchical clustering. Validation on an independent data set further illustrates the reliability of this typing. Three subtypes are characterized by divergent biological pathways and significant association with survival outcomes. In this analysis, we compare different subtypes to detect variances in pathways and also grope for the gene interaction network in the worse prognosis group. Furthermore, methylation analysis demonstrates epigenetic changes in subtypes and further validate the findings in genome and transcriptome. Our method is highly reproducible and able to identify stable categories with gene expression patterns and clinical meaning, which may be informative of tumor behavior and prognosis.

The clinical features are markedly different in survival outcome, grade and stage between subgroups. EC1 is associated with advanced grade, stage and worse prognosis but there is no significant difference between EC2 and EC3. Given the set of characteristic subtype abnormalities, we deem it likely that patients transition between subtypes during different stages of their disease. The explanation may lie in the origin that ccRCC stem from renal tubular epithelial cells and the three subtypes present similar genetic changes including loss of 3p, gain of 5q and somatic mutations or epigenetic alterations of VHL. Future studies on larger number of patients are needed to validate the cell origin and transition process of different subtypes.

Further analysis indicates the up-regulated pathways and hypomethylation genes mainly concentrate on Focal adhesion and mobility in EC1. Other pathways referring to Chemokine and cytokine also play as an assistant to produce progress. This kind of panel of genes in EC1 regulate EMT and cell cycle, causing tumor invasion and metastasis even before diagnosis and become aggressive and lethal compared with other subtypes. Early diagnosis and treatment are essential for patients with this class of molecular subtypes. The subtypes with better prognosis possess relatively overexpressed genes associated with hypoxia, PPAR signaling pathway and drug metabolism cytochrome P450. Intriguingly, these up-regulated genes or pathways are known to be broadly dysregulated in ccRCC. We have discovered VHL and other structural alterations in most samples across subtypes and reasoned that EC1 subtype may have acquired other genetic variations that enhance its ability of invasion and proliferation, contribute to a more aggressive phenotype and cover up the signature of VHL inactivation. In addition, it will be of interest to clarify the key changes that shape the unique subtype and elucidate the relationship between subtypes and treatment sensitivity.

## Conclusion

Our cross-platform molecular analyses mirror a correlation between the EC1 subtype and worsened prognosis and highlight a number of important characteristics of genetics. Further analysis identifies some critical genes that may lead to the bad clinical outcome and become prognostic biomarkers, which will hopefully provide the foundation for the development of effective forms of therapy for this disease. Our work should lay the groundwork for an improved understanding of ccRCC molecular typing and personalized therapeutic approaches that different subtypes may require.

## Additional files

Additional file 1: **Figure S1.** Using R package “ConsensusClusterPlus” to cluster GEO data and the cumulative distribution function (CDF) reaches a maximum when k = 3, thus consensus and cluster confidence is at a maximum. (PDF 1485 kb)

Additional file 2: **Table S1.** Enriched genes in Actin Cytoskeleton pathway. (XLSX 11 kb)

Additional file 3: **Table S2.** Enriched genes in MAPK Signal pathway. (XLSX 11 kb)

Additional file 4: **Table S3.** Enriched genes in Focal Adhesion pathway. (XLSX 11 kb)

Additional file 5: **Table S4.** Enriched genes in Pathways in Cancer. (XLSX 11 kb)

Additional file 6: **Table S5.** Enriched genes in Chemokine Signaling pathway. (XLSX 11 kb)

Additional file 7: **Figure S2.** Seven major modules of gene interaction network in EC1. (PDF 7 kb)

Additional file 8: **Figure S3.** Clustering dendrograms of genes, with dissimilarity based on topological overlap, together with assigned module colors. (PDF 30 kb)

Additional file 9: **Figure S4.** Coefficient between modules and clinical parameters. Pvalue is below coefficient value. (PDF 35 kb)

Additional file 10: **Figure S5.** Weighted Gene Co-expression Network plot of blue module. Red color means hub gene and the thickness of the line represents the connect strength of the interaction. Circle size: the number of connectivity. (PDF 607 kb)

Additional file 11: **Figure S6.** Boxplot of mean methylation of EC1 and EC2–3. (PDF 155 kb)

Additional file 12: Supplementary material and methods. (DOCX 19 kb)

## Abbreviations

ccRCC: Clear Cell Renal Cell Carcinoma.
EC: Expression clusters.
EMT: Epithelial-to-mesenchyme transition.
HM450K: Human Methylation 450 K Bead Chip.
SCNA: Somatic copy number analysis.
WGCNA: Weighted Correlation Network analysis
