# Supplementary figures and images for: Integrated genomic analysis identifies clinically relevant subtypes of renal clear cell carcinoma
Source: BMC Cancer. 2018 Mar 13;18:287. doi: 10.1186/s12885-018-4176-1 (PMC5851245; doi:10.1186/s12885-018-4176-1)

**k=2**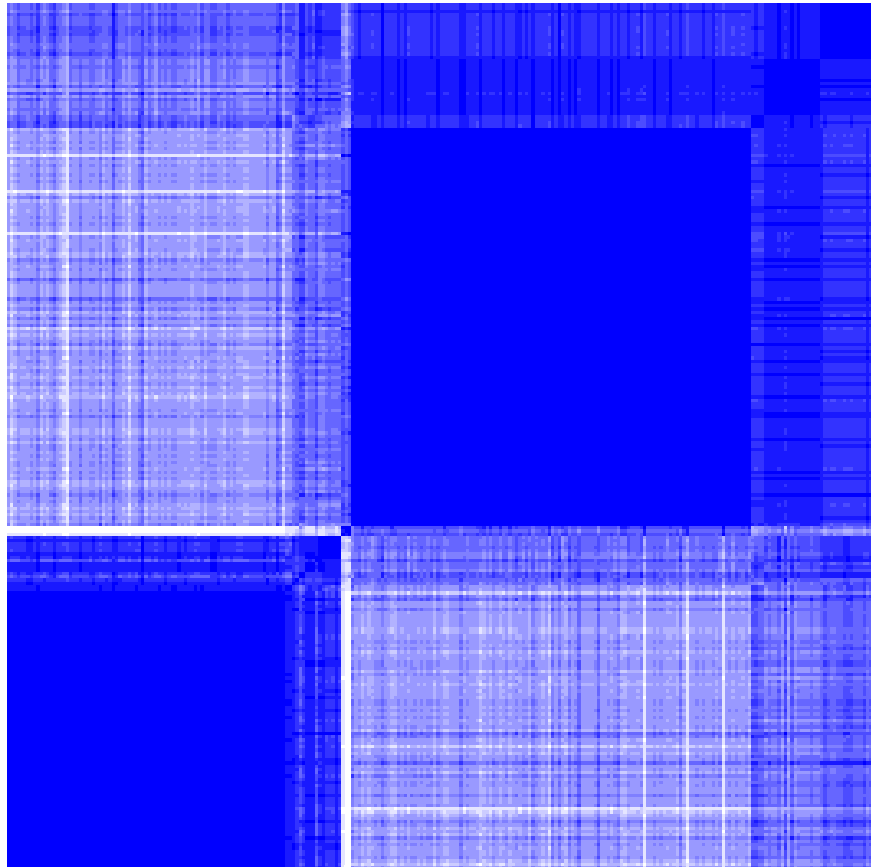**k=3**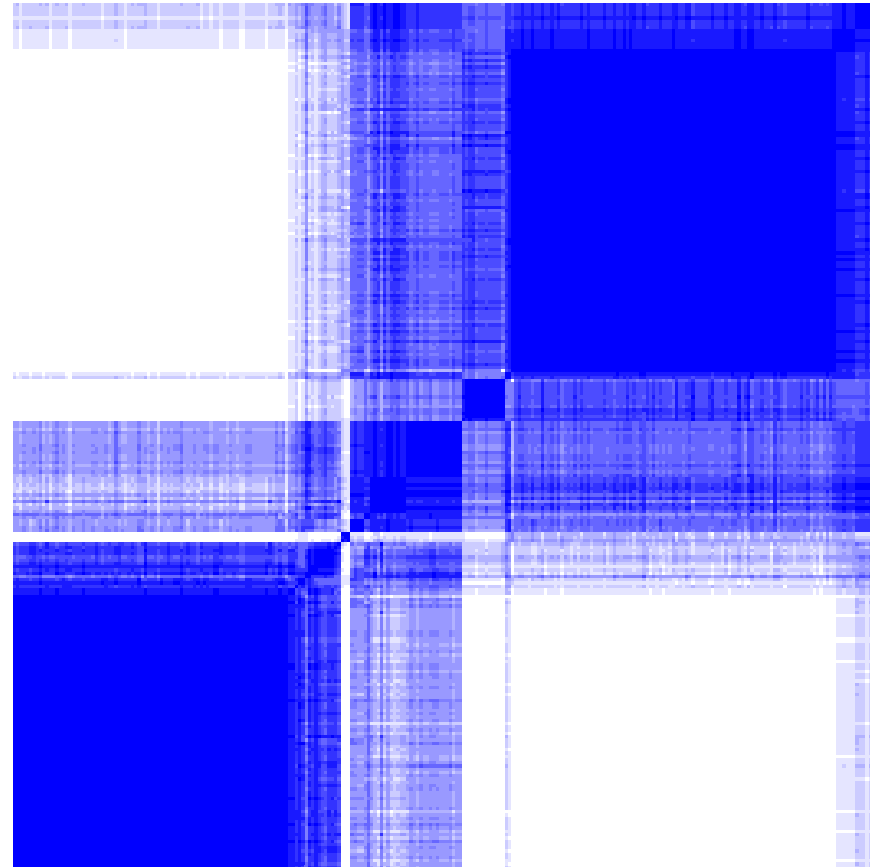**k=4**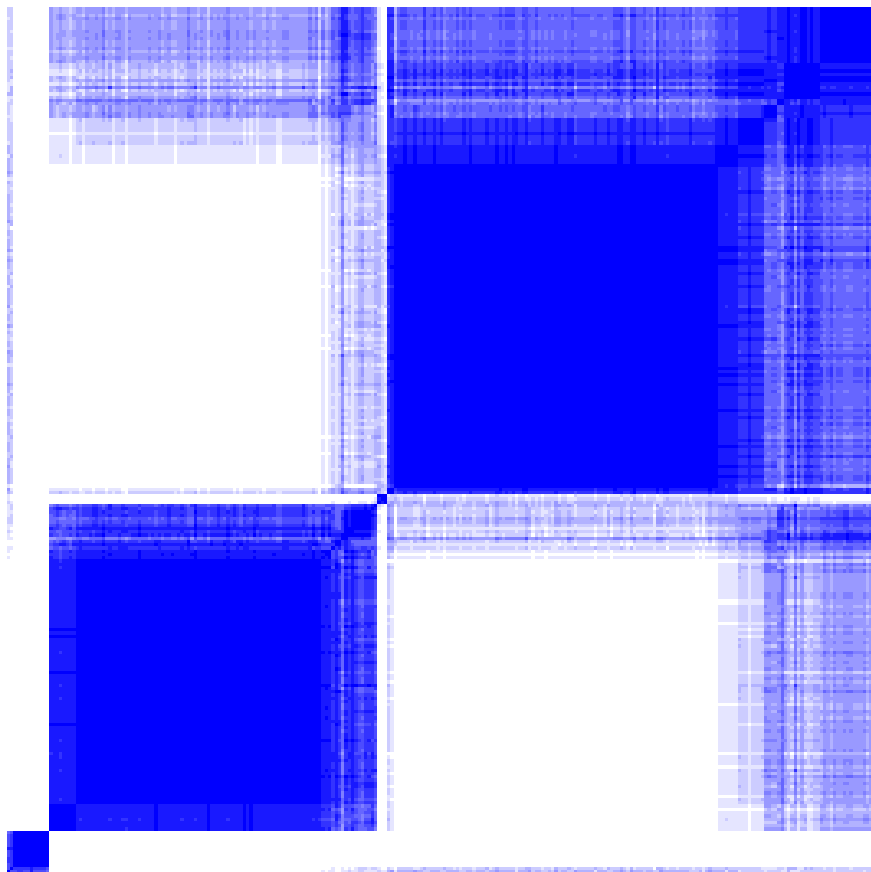**k=5**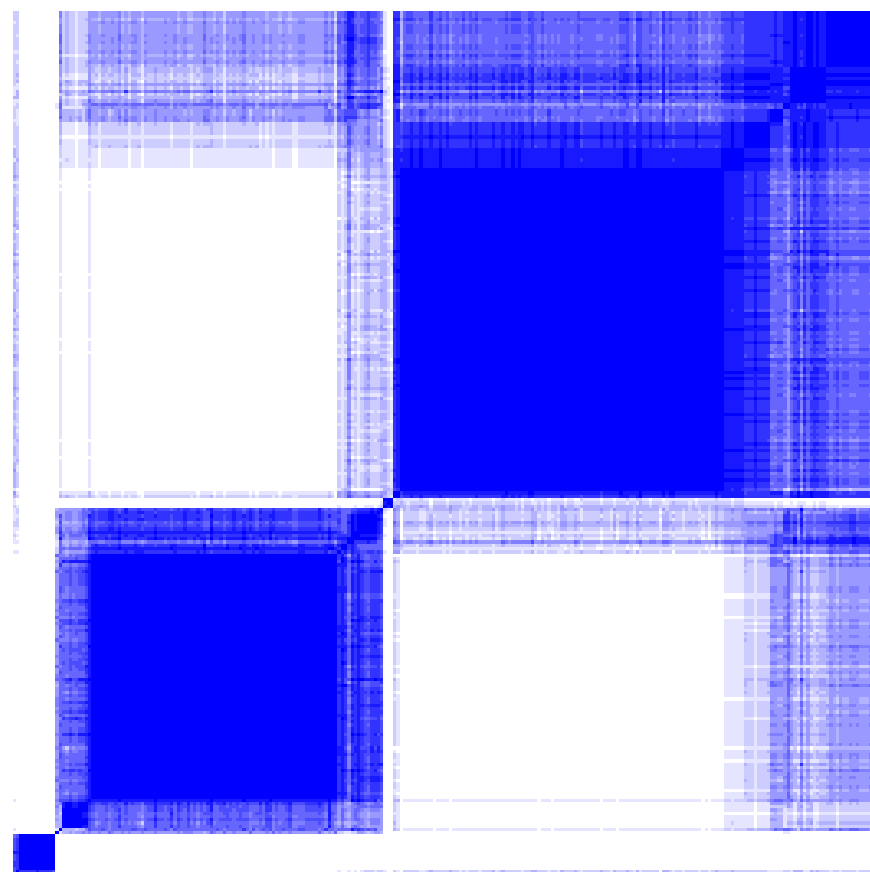**consensus CDF**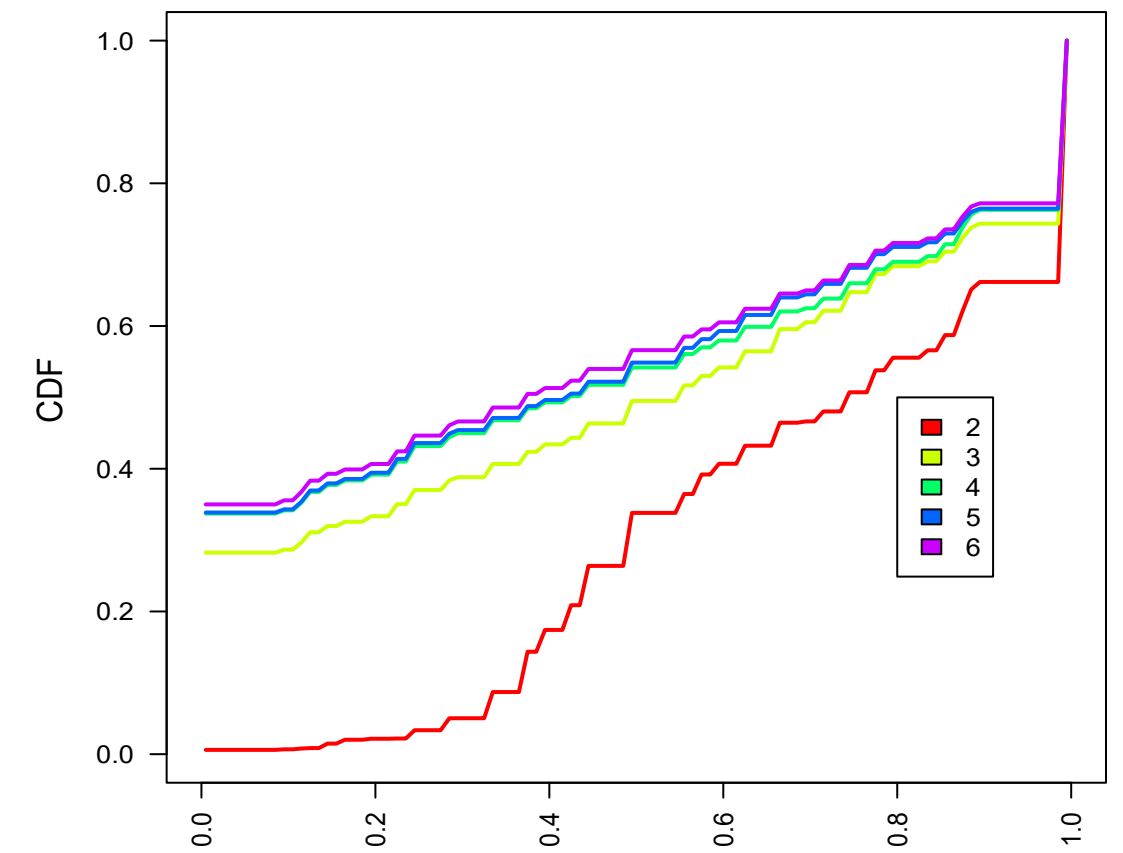**consensus index****Delta area**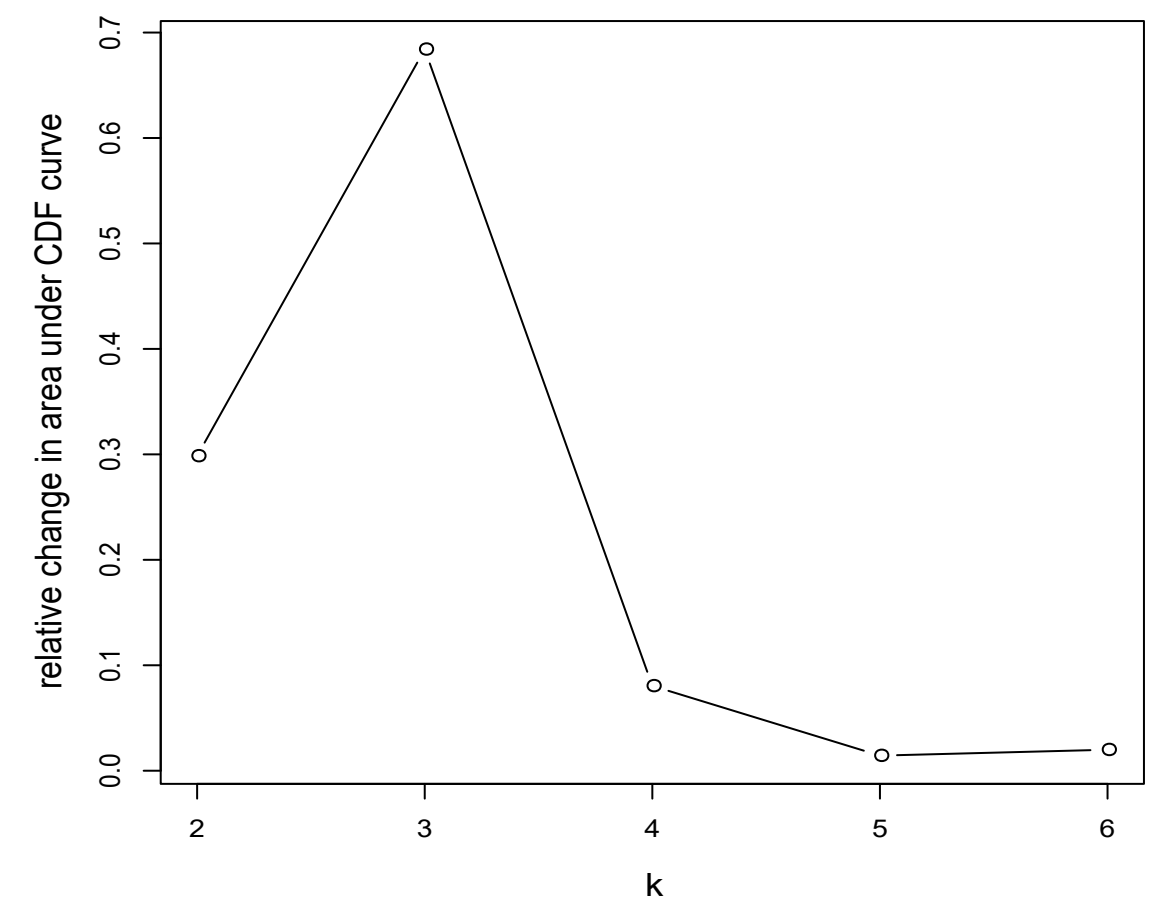

Supplement: Supplementary file 1 — Figure S1. Using R package “ConsensusClusterPlus” to cluster GEO data and the cumulative distribution function (CDF) reaches a maximum when k = 3, thus consensus and cluster confidence is at a maximum. (PDF 1485 kb) [file 12885_2018_4176_MOESM1_ESM.pdf]

## Clustering of module eigengenes

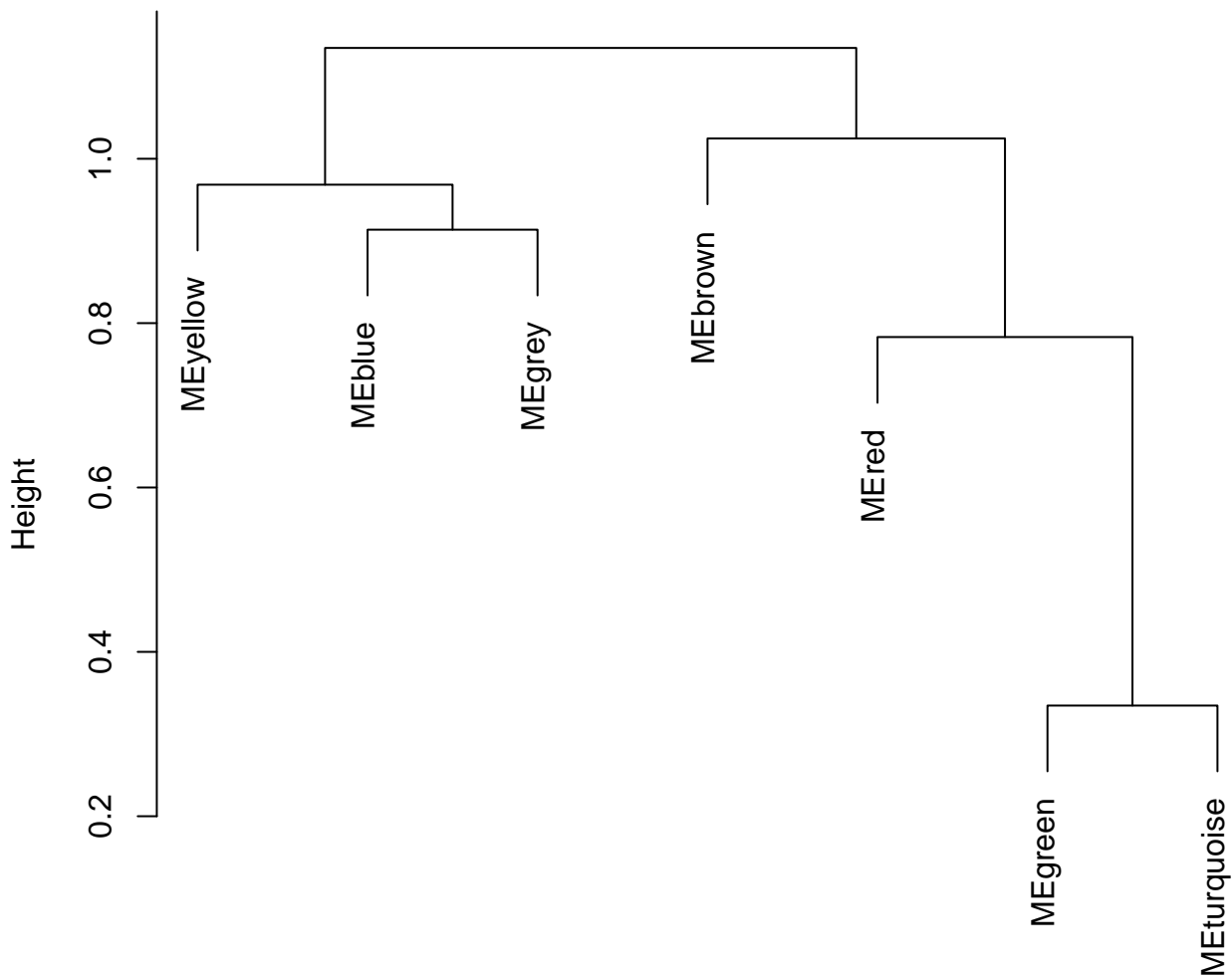

Supplement: Supplementary file 7 — Figure S2. Seven major modules of gene interaction network in EC1. (PDF 7 kb) [file 12885_2018_4176_MOESM7_ESM.pdf]

# Gene dendrogram and module colors

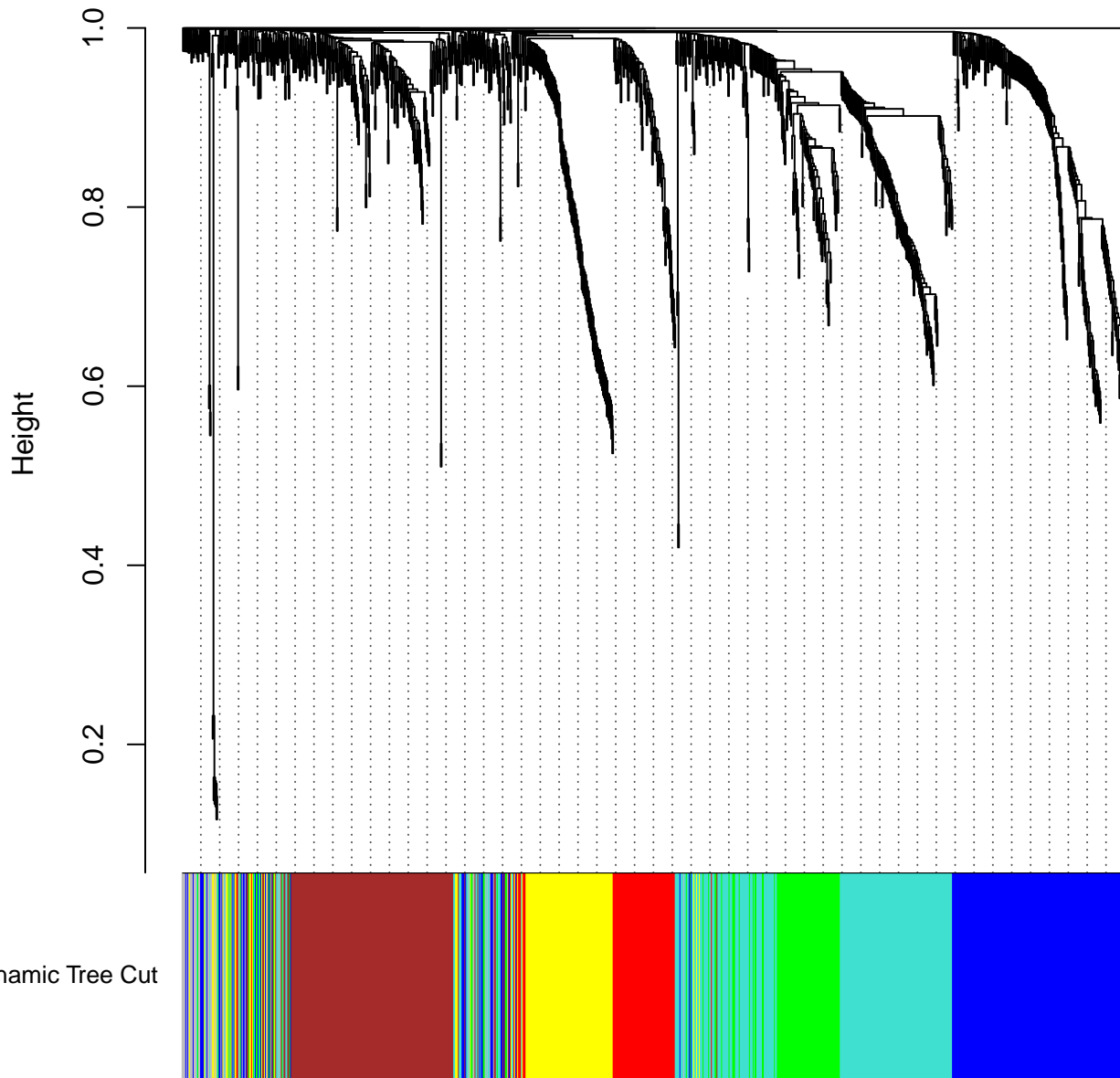

Supplement: Supplementary file 8 — Figure S3. Clustering dendrograms of genes, with dissimilarity based on topological overlap, together with assigned module colors. (PDF 30 kb) [file 12885_2018_4176_MOESM8_ESM.pdf]

# Module-trait relationships

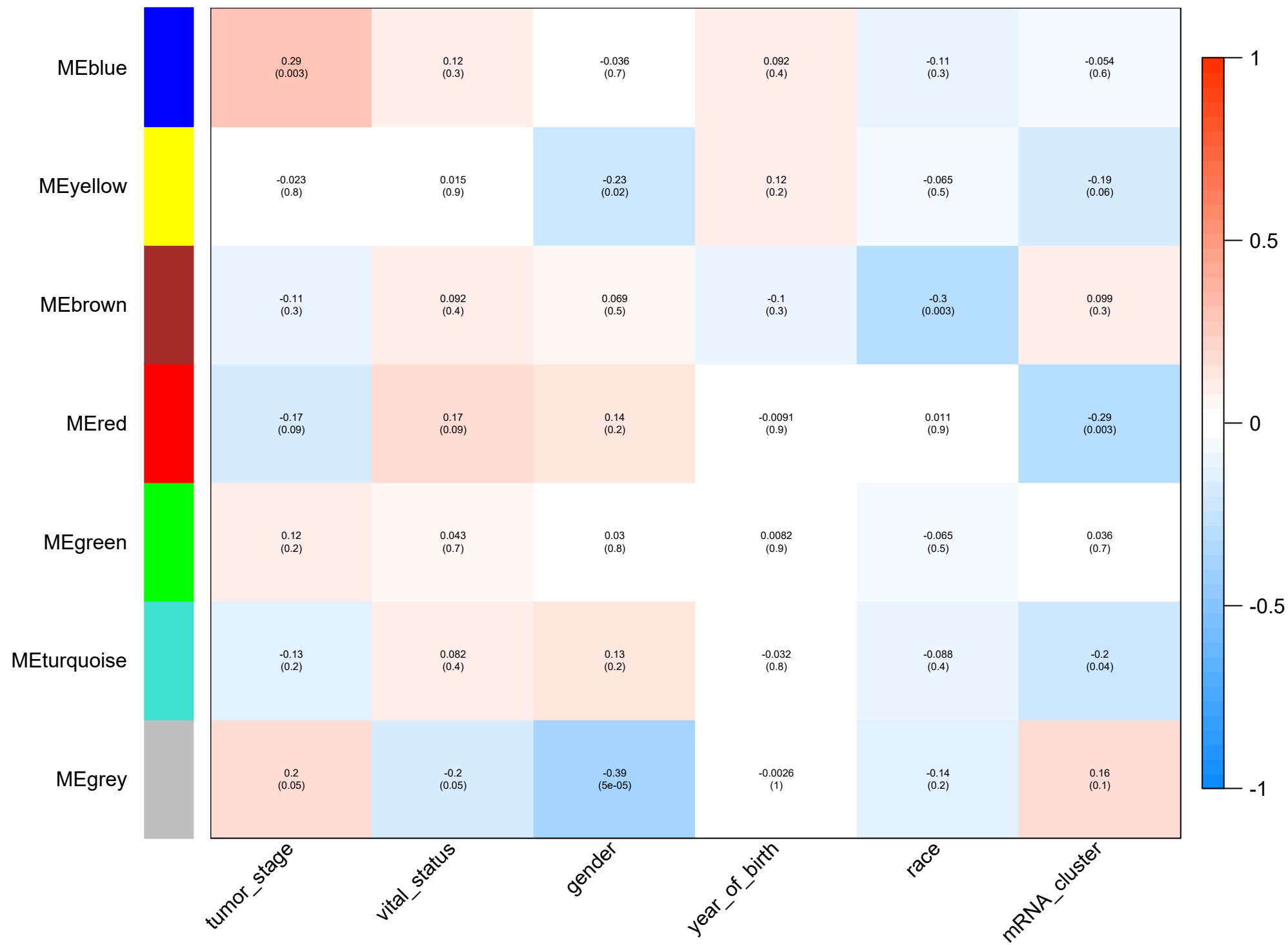

Supplement: Supplementary file 9 — Figure S4. Coefficient between modules and clinical parameters. Pvalue is below coefficient value. (PDF 35 kb) [file 12885_2018_4176_MOESM9_ESM.pdf]

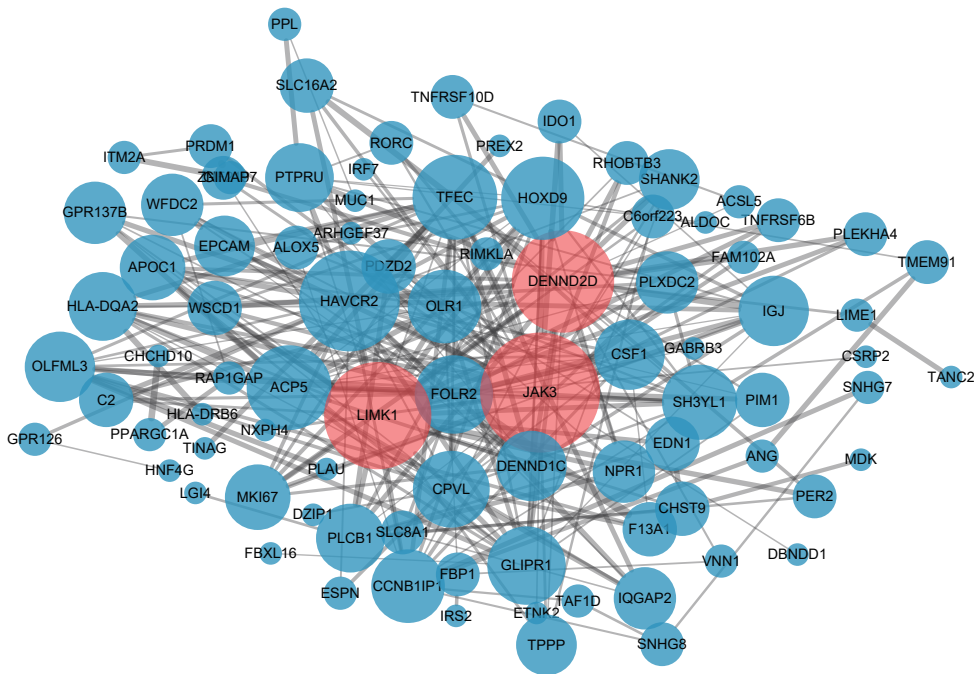

Supplement: Supplementary file 10 — Figure S5. Weighted Gene Co-expression Network plot of blue module. Red color means hub gene and the thickness of the line represents the connect strength of the interaction. Circle size: the number of connectivity. (PDF 607 kb) [file 12885_2018_4176_MOESM10_ESM.pdf]

# Mean DNA methylation

Treatment

EC1 EC2-3

Mean DNA methylation ( $\beta$ -values)

0.52  
0.48  
0.44  
0.40

P-value = 3.7e-01

EC1

EC2-3

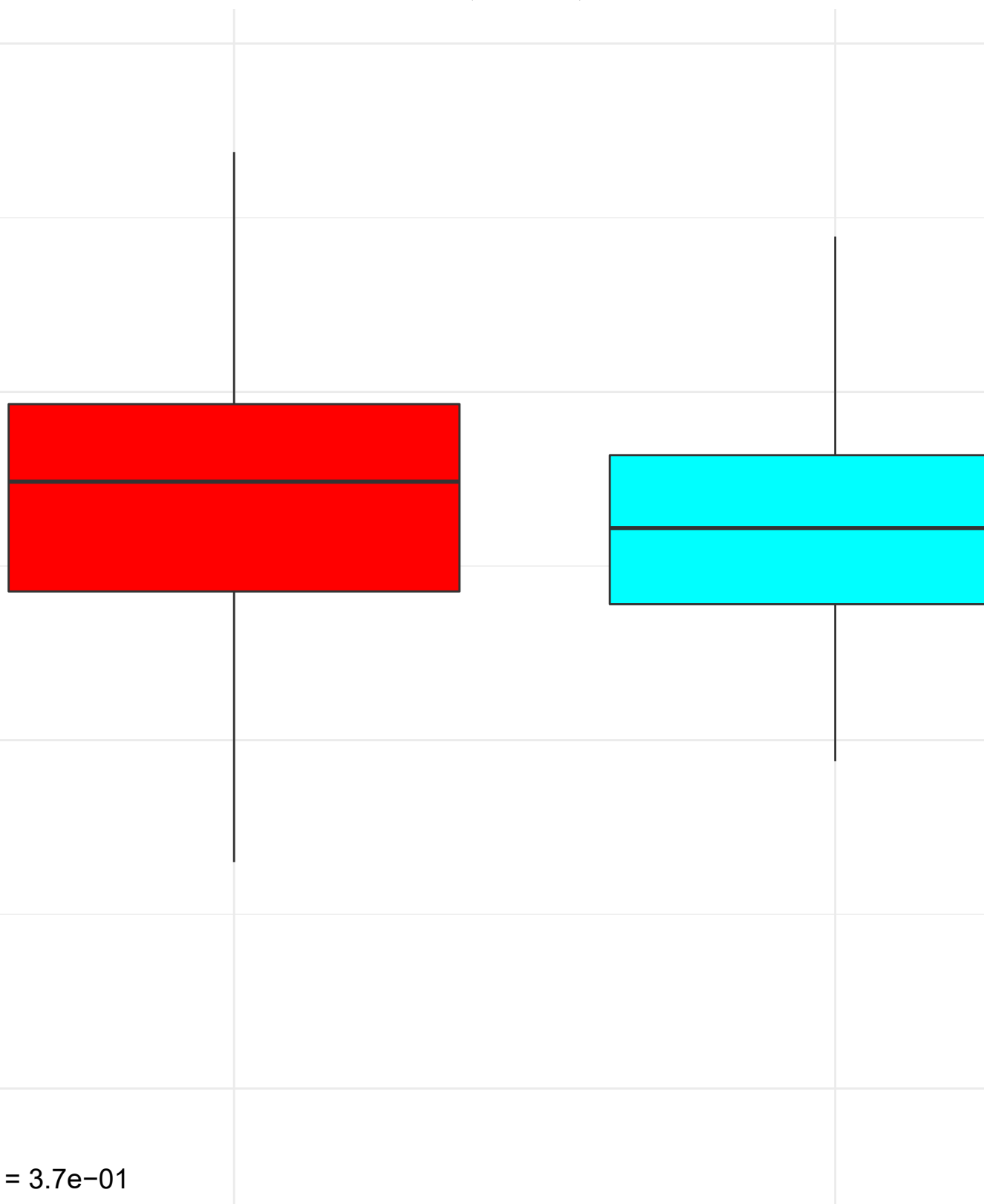

Supplement: Supplementary file 11 — Figure S6. Boxplot of mean methylation of EC1 and EC2–3. (PDF 155 kb) [file 12885_2018_4176_MOESM11_ESM.pdf]
