# Supplementary material for: Integrated genomic analysis identifies clinically relevant subtypes of renal clear cell carcinoma
Source: BMC Cancer. 2018 Mar 13;18:287. doi: 10.1186/s12885-018-4176-1 (PMC5851245; doi:10.1186/s12885-018-4176-1)
Supplement: Supplementary file 12 — Supplementary material and methods. (DOCX 19 kb) [file 12885_2018_4176_MOESM12_ESM.docx]

## Supplementary material and methods

**Patients and tumor Samples.**

Level 3 data of 537 samples including somatic variants (SNVs and SCNAs), methylation HMK450 and expression data can be obtained from Genomic Data Commons (GDC) data portal (<http://gdc-portal.nci.nih.gov>). Gene expression data of 265 ccRCC patients from the validation set were collected from GEO (GSE73731) (<https://www.ncbi.nlm.nih.gov/geo/>).

**Data Filtering**

We apply two filters described by R package “genefilter” to eliminate unreliably measured genes and to limit the clustering to relevant genes. The first filter removed genes with low variability across patients. The second filter eliminated genes with out of the bound of coefficient of variation we set, resulting in 1,017 genes.

**Identification of expression-based subtypes**

We apply consensus clustering which has been used widely to discover molecular subtypes with hierarchical clustering to cluster ccRCC expression data. The distance matrix was 1 minus the Pearson’s correlation coefficient, and the procedure was repeated over 1000 iterations and a subsampling ratio of 0.6, using the 441 ccRCC samples and two normal samples and 1,017 reliably expressed genes. The cluster number was estimated by computing the CDF for each k and area under the CDF curve.

**Identify marker genes for each cluster**

For each gene that passes filter, a binary classifier is constructed based on the mean cluster-expression level. The AUROC (area under the receiver operating curve) is used to quantify the accuracy of the prediction. A *P*-value is assigned to each gene using the Wilcoxon signed-rank test, comparing gene ranks in the cluster with the highest mean expression with all others. Genes with AUROC > 0.8 together with *P* < 0.01 are defined as marker genes. The AUROC threshold corresponds to the 99^th^ percentile of the AUROC distributions obtained from 100 random permutations of cluster labels for ccRCC expression data.

**Gene set enrichment analysis**

Support that expression data set D with N genes and k samples. The predefined gene set is S of $\boldsymbol{N}_{\boldsymbol{H}}$ genes. The goal of GSEA is to determine whether the members of S are randomly distributed throughout L or primarily found at the top or bottom.

$$\boldsymbol{P}_{\boldsymbol{hit}}\left( \boldsymbol{S},\boldsymbol{i} \right)=\sum_{\begin{aligned} \boldsymbol{g}_{\boldsymbol{j}}\in\boldsymbol{S} \\ \boldsymbol{j}\leq\boldsymbol{i} \end{aligned}} \frac{{|\boldsymbol{r}_{\boldsymbol{j}}|}^{\boldsymbol{p}}}{\boldsymbol{N}_{\boldsymbol{R}}}, \boldsymbol{where} \boldsymbol{N}_{\boldsymbol{R}}=\sum_{\boldsymbol{g}_{\boldsymbol{i}}\in\boldsymbol{S}} {{|\boldsymbol{r}}_{\boldsymbol{j}}|}^{\boldsymbol{p}}$$

$$\boldsymbol{P}_{\boldsymbol{miss}}\left( \boldsymbol{S},\boldsymbol{i} \right)=\sum_{\begin{aligned} \boldsymbol{g}_{\boldsymbol{j}}\in\boldsymbol{S} \\ \boldsymbol{j}\leq\boldsymbol{i} \end{aligned}} \frac{{|\boldsymbol{r}_{\boldsymbol{j}}|}^{\boldsymbol{p}}}{|\boldsymbol{N}-\boldsymbol{N}_{\boldsymbol{H}}|}$$

ES is the maximum deviation from zero of $\boldsymbol{P}_{\boldsymbol{hit}}-\boldsymbol{P}_{\boldsymbol{miss}}$. Ranking procedure to produce gene list $\mathbf{L}=\{\boldsymbol{g}_{\mathbf{1}},\cdots,\boldsymbol{g}_{\boldsymbol{N}}\}$ includes a correlation (or other ranking metric) and a phenotype or profile of interest C. Genes are ranked according to the correlation: $\mathbf{r}\left( \boldsymbol{g}_{\boldsymbol{j}} \right)=\boldsymbol{r}_{\boldsymbol{j}}$, of their expression profiles with C. An exponent p to control the weight of the step. We set $\mathbf{p}=\mathbf{1}$ in our data and the significance is assessed by permutation test. If the gene contributes to the leading-edge subset within the gene set, it would be defined as core enrichment gene (Top 30%).

**Differential expression genes analysis**

We used R package “DEseq2” to analyze differential genes(DEs) and set $\left| \boldsymbol{log}_{\mathbf{2}}\boldsymbol{FC} \right|\geq\mathbf{2}, \boldsymbol{P}_{\boldsymbol{adj}}<\mathbf{0}.\mathbf{01}$(EC1/EC2) as thresholds for DEs.

The differential expression analysis in DESeq2 uses a generalized linear model of the form:

$$\boldsymbol{K}_{\boldsymbol{ij}}\sim\boldsymbol{NB}(\boldsymbol{\mu}_{\boldsymbol{ij}},\boldsymbol{\alpha}_{\boldsymbol{i}})$$

$$\boldsymbol{\mu}_{\boldsymbol{ij}}=\boldsymbol{s}_{\boldsymbol{j}}\boldsymbol{q}_{\boldsymbol{ij}}$$

$$\boldsymbol{log}_{\mathbf{2}}\left( \boldsymbol{q}_{\boldsymbol{ij}} \right)=\boldsymbol{\chi}_{\boldsymbol{j}}\boldsymbol{\beta}_{\boldsymbol{i}}$$

where counts $\boldsymbol{K}_{\boldsymbol{ij}}$ for gene $\mathbf{i}$ sample $\mathbf{j}$ are modeled using a negative binomial distribution with fitted mean $\boldsymbol{\mu}_{\boldsymbol{ij}}$ and a gene-specific dispersion parameter $\boldsymbol{\alpha}_{\boldsymbol{i}}$. The fitted mean is composed of a sample-specific size factor $\boldsymbol{s}_{\boldsymbol{j}}$ and a parameter $\boldsymbol{q}_{\boldsymbol{ij}}$ proportional to the expected true concentration of fragments for sample $\mathbf{j}$. The coefficients $\boldsymbol{\beta}_{\boldsymbol{i}}$ give the log2 fold changes for gene $\mathbf{i}$ for each column of the model matrix $\boldsymbol{\chi}$.

**weighted correlation network analysis**

The WGCNA algorithm first assumes that the genetic network obeys the scale-free distribution and defines the gene co-expression correlation matrix, the adjacency function formed by the genetic network, and then calculates the dissimilarity coefficients of the different nodes and constructs the hierarchical clustering tree. Different branches of the cluster tree represent different gene modules (modules). The gene expression within the module is high, and the scores of different modules are low. WGCNA algorithm is widely used to explore the relationship between the module and a specific phenotype or disease, and ultimately identify the target gene of disease treatment, gene network purposes.

Procedure:

1. Construct gene co-expression correlation matrix, correlation between gene m and n is $S_{mn}=|cor(m,n)|$
2. Define adjacency function: $a_{mn}=power\left( S_{mn},\beta\right)={|S_{mn}|}^{\beta}$, $\beta$ is adjacency function coefficient.
3. Transform correlation matrix $S=\left[ S_{mn} \right]$ into adjacency matrix $A=\left[ a_{mn} \right]$. After $\beta$ is confirmed, adjacency matrix is changed to topological matrix $\Omega=\left[ \omega_{mn} \right]$, which $\omega_{mn}=\frac{l_{mn}+a_{mn}}{\min\left\{ k_{m},k_{n} \right\}+l-a_{mn}}$. Formula $l_{mn}=\sum_{\mu} a_{m\mu}a_{\mu n}$ represents the sum of the adjacency coefficients of the nodes that connect with both gene m and n, $k_{m}=\sum_{\mu} a_{m\mu}$ is the sum of adjacency coefficients of nodes that connect with gene m. Similarly, $k_{n}=\sum_{\mu} a_{n\mu}$ is for gene n. The degree of dissimilarity of the nodes is measured by $d_{mn}^{\omega}=1-\omega_{mn}$.
4. Construct hierarchical clustering tree based on the dissimilar coefficient $d_{mn}^{\omega}$ between genes and identify gene modules.

**Differentially methylated regions Analysis**

To find differentially regions between subtypes we use the methylation beta-values (ranging from 0.0 to 1.0).

Firstly, the difference was computed between the mean methylation of each group for each probe. Secondly, we calculate the p-value using the wilcoxon test using the Benjamini-Hochberg adjustment method. A minimum absolute beta values delta of 0.2 and a false discovery rate (FDR)-adjusted Wilcoxon rank-sum P-value of < 0.01 are set as for the difference.
